# Supplementary figures and images for: HMGB1-activatied NLRP3 inflammasome induces thrombocytopenia in heatstroke rat
Source: PeerJ. 2022 Aug 4;10:e13799. doi: 10.7717/peerj.13799 (PMC9357367; doi:10.7717/peerj.13799)

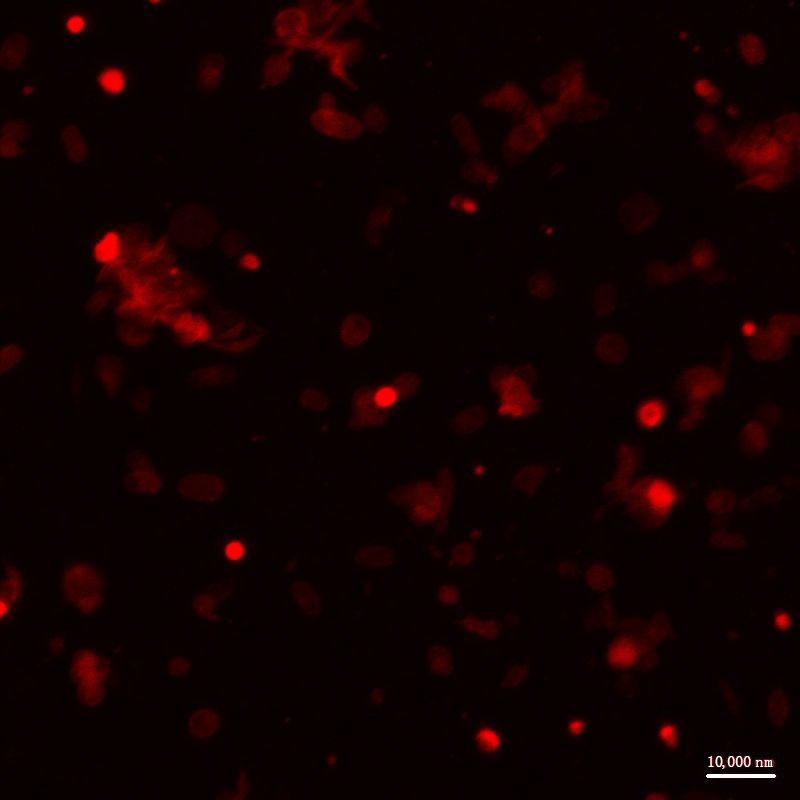

Supplement: Figure S2 [file peerj-10-13799-s002.zip › Fig.2/Fig.2C raw images of NLRP3 Inflammasome assembly in platelets of rat at 9 h after HS onset/HS-9h ASC.tif]

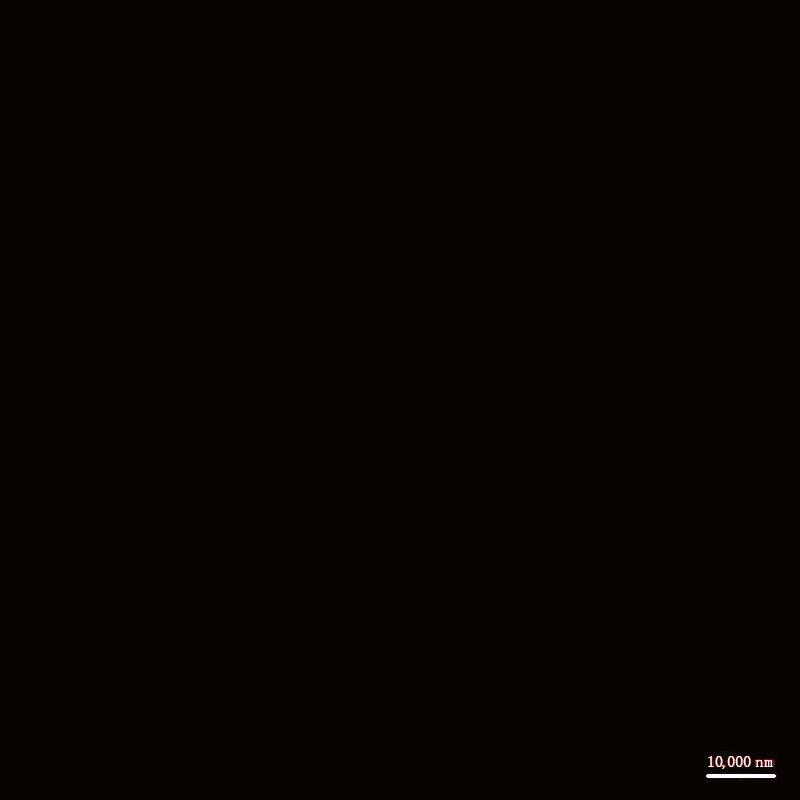

Supplement: Figure S2 [file peerj-10-13799-s002.zip › Fig.2/Fig.2C raw images of NLRP3 Inflammasome assembly in platelets of rat at 9 h after HS onset/HS-9h DAPI.tif]

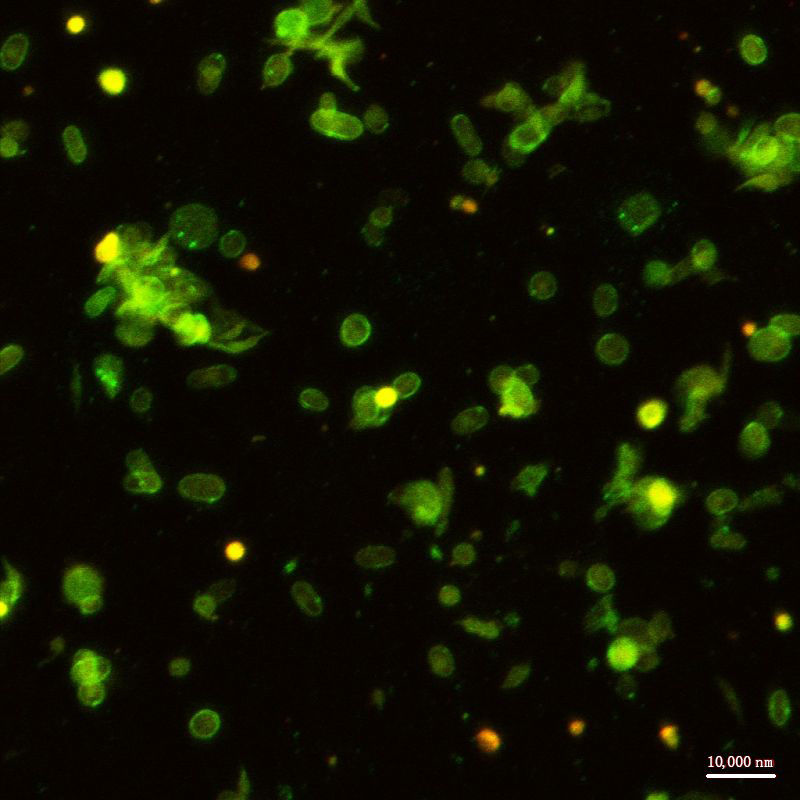

Supplement: Figure S2 [file peerj-10-13799-s002.zip › Fig.2/Fig.2C raw images of NLRP3 Inflammasome assembly in platelets of rat at 9 h after HS onset/HS-9h Merge.tif]

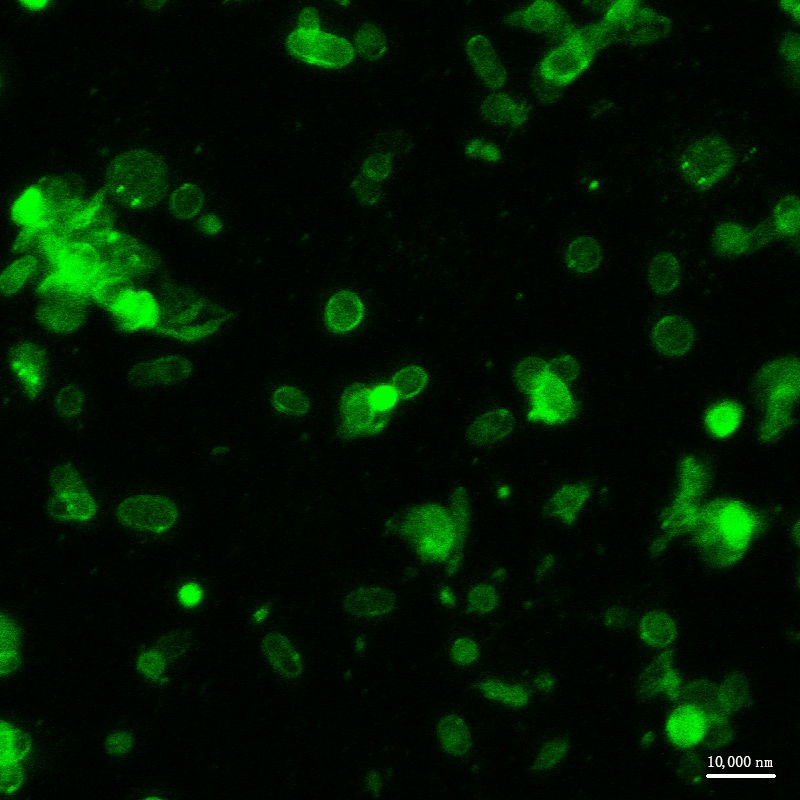

Supplement: Figure S2 [file peerj-10-13799-s002.zip › Fig.2/Fig.2C raw images of NLRP3 Inflammasome assembly in platelets of rat at 9 h after HS onset/HS-9h NLRP3.tif]

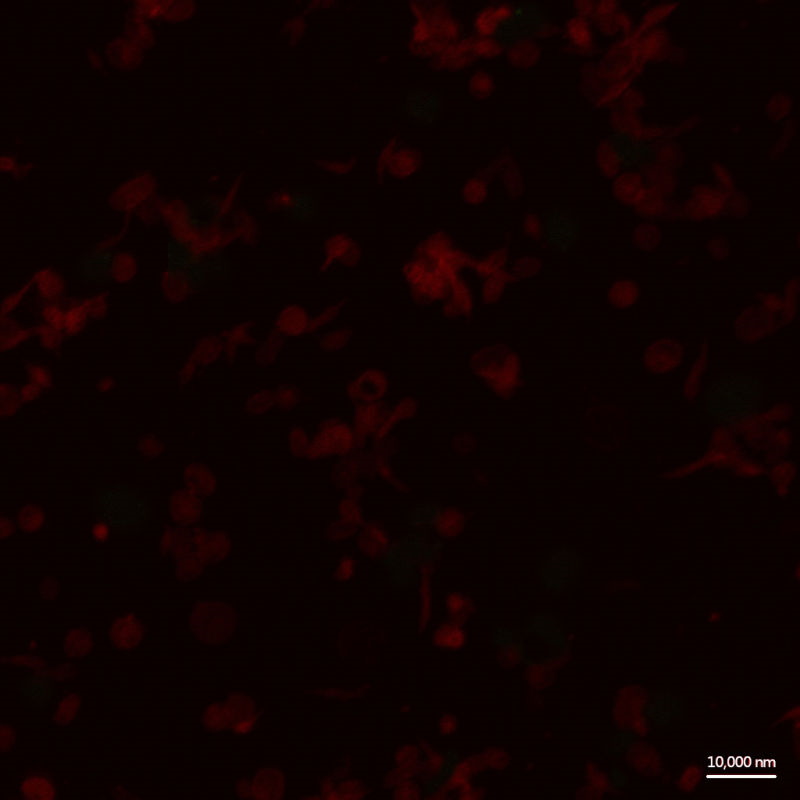

Supplement: Figure S2 [file peerj-10-13799-s002.zip › Fig.2/Fig.2C raw images of NLRP3 Inflammasome assembly in platelets of rat at 9 h after HS onset/Sham ASC.tif]

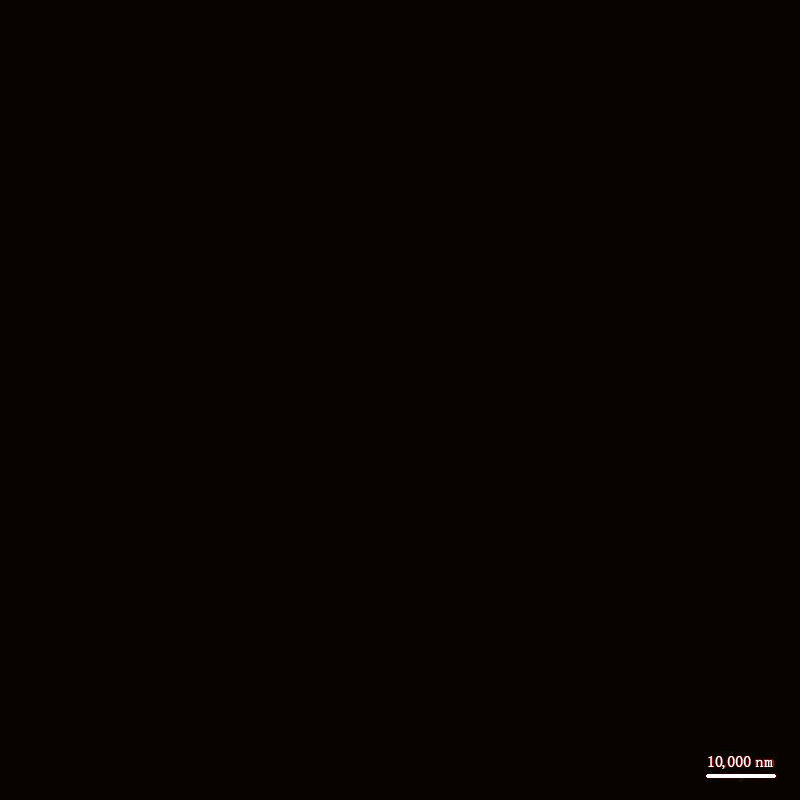

Supplement: Figure S2 [file peerj-10-13799-s002.zip › Fig.2/Fig.2C raw images of NLRP3 Inflammasome assembly in platelets of rat at 9 h after HS onset/Sham DAPI.TIF]

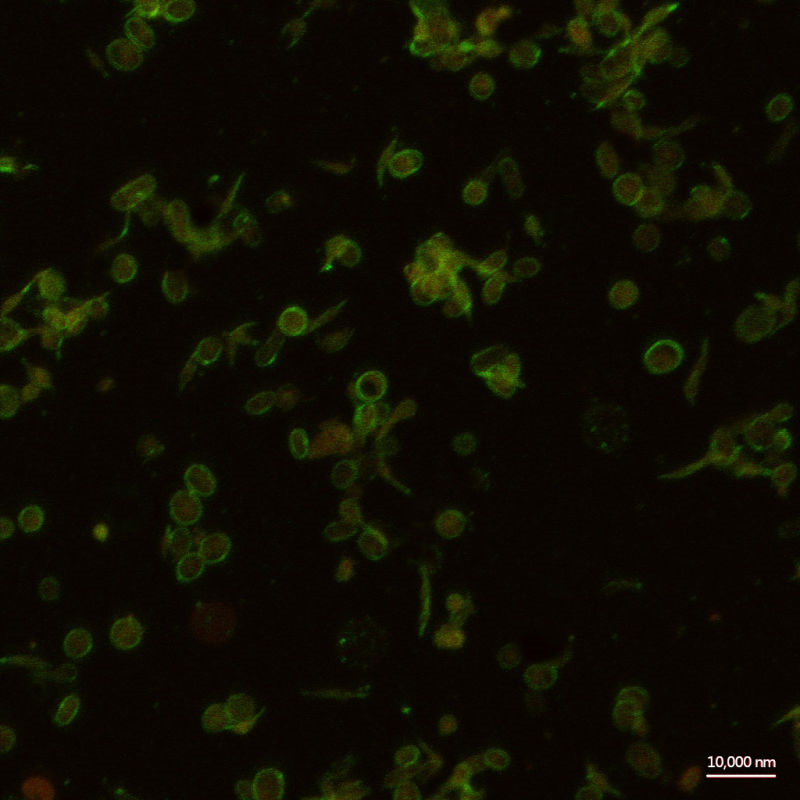

Supplement: Figure S2 [file peerj-10-13799-s002.zip › Fig.2/Fig.2C raw images of NLRP3 Inflammasome assembly in platelets of rat at 9 h after HS onset/Sham Merge.tif]

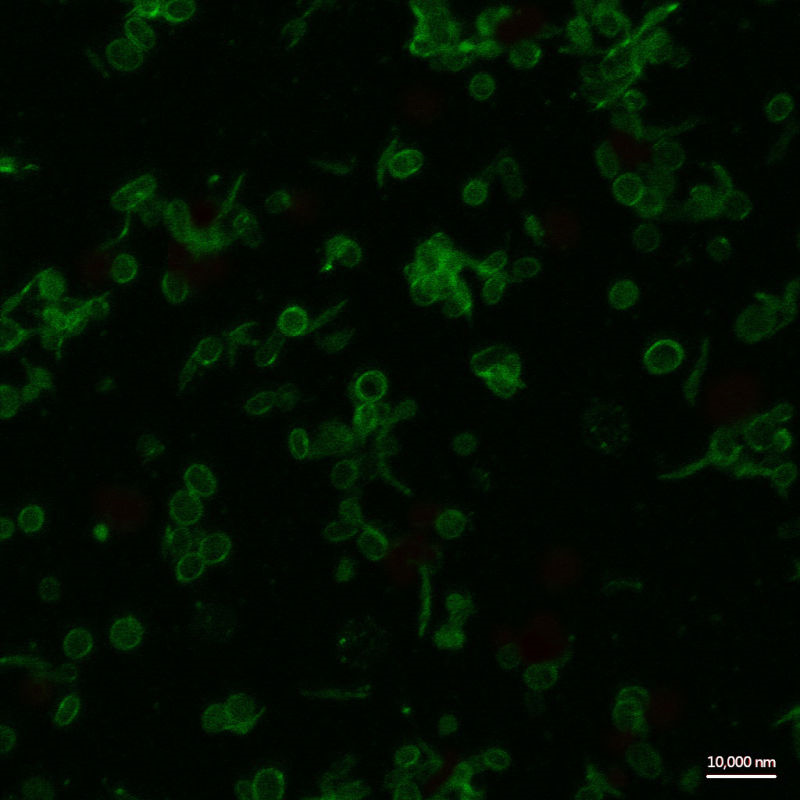

Supplement: Figure S2 [file peerj-10-13799-s002.zip › Fig.2/Fig.2C raw images of NLRP3 Inflammasome assembly in platelets of rat at 9 h after HS onset/Sham NLRP3.tif]

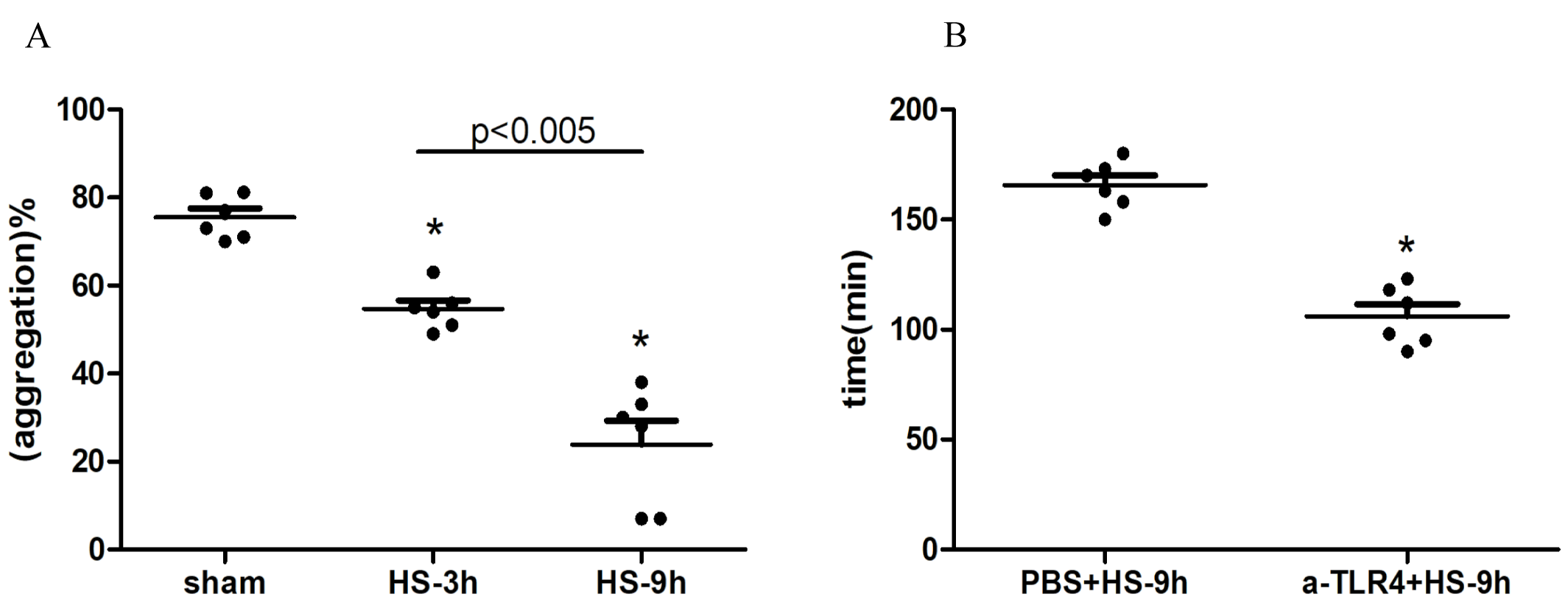

Supplement: Supplemental Information 6 — (A). Changes of platelet aggregation in HS rats (n=6). *p ¡ 0.005 vs sham. (B). Changes of heat exposure time in HS rats (n=6). *p ¡ 0.001 vs sham. [file peerj-10-13799-s006.png]
